# Supplementary material for: SESNet: sequence-structure feature-integrated deep learning method for data-efficient protein engineering
Source: J Cheminform. 2023 Feb 3;15:12. doi: 10.1186/s13321-023-00688-x (PMC9898993; doi:10.1186/s13321-023-00688-x)
Supplement: Supplementary file 1 — Additional file 1: Table S1. Spearman correlation in Figure 2A. Comparison to other supervised and unsupervised models for fitness prediction on the single-site mutants of 20 datasets. The one marked in bold denotes the best performance. Table S2. Spearman correlation in Figure 2B. Fitness prediction of double-site mutants by unsupervised models (ESM-IF1, ESM-1v and MSA transformer), or supervised models (ECNet and ESM-1b) and SESNet trained on the data of single-site mutants. The one marked in bold denotes the best performance. Table S3. Spearman correlation in Figure 2C. Prediction of quadruple variants of avGFP using models trained on single, double, triple-site mutants and all the above three. Table S4. Detailed information on the proteins listed in the dataset of Tables 1-3. The protein fitness classification and the number of sites being mutated of each protein. Table S5. Ablation study results. Ablation study was performed in the testing when we removed each of the three modules in the integrated model. The average spearman correlation of all datasets shows that model including all the three components are the most accurate, and all three parts contribute positively to the performance of the integrated model, with the global encoder contributing the most. Table S6. Ablation study of the pre-trained model tested on GFP datasets. The spearman correlation was predicted by our models which is pre-trained on single-site and numerous double-sites variants generated by the unsupervised model ESM-IF1. Table S7. Hyperparameter configurations for different dataset. Figure S1. Attention score of sites on the wildtype sequence. Attention scores of sites generated by SESNet (A) and the model without the structure module (B) trained on the 1084 single-site mutants of the dataset of GFP. We picked up the top 20 attention-score AA sites predicted by SESNet with and without structure module, respectively. When the structural module is present, there are five sites (marked by [file 13321_2023_688_MOESM1_ESM.docx]

**Additional file**

**SESNet: sequence-structure feature-integrated deep learning method for data-efficient protein engineering**

Table S1. **Spearman correlation in Figure 2A.** Comparison to other supervised and unsupervised models for fitness prediction on the single-site mutants of 20 datasets. The one marked in bold denotes the best performance.

|  | ESM-IF1 | ESM-1v | MSA Transformer | ESM-1b | ECNet | SESNet |
| --- | --- | --- | --- | --- | --- | --- |
| BLAT_ECOLX | 0.673 | 0.692 | 0.538 | 0.847 | 0.826 | **0.903** |
| P84126_THETH | 0.519 | 0.564 | 0.656 | 0.844 | 0.792 | **0.858** |
| RL40B_YEAST | 0.372 | 0.365 | 0.647 | 0.809 | 0.802 | **0.872** |
| TIM_SULSO | 0.506 | 0.617 | 0.613 | 0.772 | 0.737 | **0.790** |
| TRPC_THEMA | 0.392 | 0.488 | 0.462 | 0.767 | 0.727 | **0.783** |
| YAP1_HUMAN | 0.485 | 0.361 | -0.007 | 0.731 | 0.670 | **0.775** |
| DLG4_RAT | 0.468 | 0.531 | 0.224 | 0.676 | 0.737 | **0.748** |
| BG_STRSQ | 0.665 | 0.670 | 0.727 | 0.530 | 0.740 | **0.747** |
| IF1_ECOLI | 0.337 | 0.356 | 0.363 | 0.719 | 0.630 | **0.726** |
| PTEN_HUMAN | 0.559 | 0.458 | 0.366 | 0.688 | 0.684 | **0.715** |
| UBC9_HUMAN | 0.485 | 0.518 | 0.425 | 0.643 | 0.690 | **0.710** |
| RASH_HUMAN | 0.070 | 0.131 | 0.089 | 0.631 | 0.599 | **0.656** |
| AMIE_PSEAE | 0.295 | 0.537 | 0.523 | 0.599 | 0.526 | **0.632** |
| SUMO1_HUMAN | 0.543 | 0.548 | 0.565 | 0.593 | 0.605 | **0.609** |
| TPMT_HUMAN | 0.560 | 0.531 | 0.530 | 0.533 | 0.541 | **0.579** |
| GAL4_YEAST | 0.326 | 0.476 | 0.386 | 0.550 | 0.514 | **0.561** |
| MK01_HUMAN | 0.155 | 0.164 | 0.182 | 0.410 | 0.375 | **0.491** |
| KKA2_KLEPN | 0.204 | 0.198 | 0.153 | 0.405 | 0.370 | **0.446** |
| MTH3_HAEAESTABILIZED | 0.423 | 0.488 | **0.564** | 0.350 | 0.419 | 0.430 |
| B3VI55_LIPST | 0.291 | 0.272 | 0.316 | 0.369 | 0.351 | **0.412** |
| AVERAGE | 0.416 | 0.448 | 0.416 | 0.623 | 0.617 | **0.672** |

Table S2. **Spearman correlation in Figure 2B.** Fitness prediction of double-site mutants by unsupervised models (ESM-IF1, ESM-1v and MSA transformer), or supervised models (ECNet and ESM-1b) and SESNet trained on the data of single-site mutants. The one marked in bold denotes the best performance.

|  | ESM-IF1 | ESM-1v | MSA Transformer | ESM-1b | ECNet | SESNet |
| --- | --- | --- | --- | --- | --- | --- |
| FOSJUN | 0.532 | 0.464 | 0.366 | 0.927 | 0.855 | **0.927** |
| TDP43 | 0.158 | 0.026 | 0.117 | 0.914 | 0.865 | **0.924** |
| GB1 | 0.337 | 0.105 | 0.329 | 0.872 | 0.830 | **0.907** |
| RRM | 0.443 | 0.536 | 0.509 | 0.877 | 0.743 | **0.881** |
| WW | 0.415 | 0.399 | 0.441 | 0.701 | 0.692 | **0.733** |
| GFP | 0.455 | 0.010 | 0.386 | 0.562 | 0.416 | **0.598** |
| AVERAGE | 0.390 | 0.257 | 0.358 | 0.809 | 0.733 | **0.828** |

Table S3. **Spearman correlation in Figure 2C.** Prediction of quadruple variants of avGFP using models trained on single, double, triple-site mutants and all the above three.

|  | ESM-1b | ECNet | SESNet |
| --- | --- | --- | --- |
| single | 0.770 | 0.512 | **0.772** |
| double | 0.847 | 0.802 | **0.850** |
| triple | 0.852 | 0.824 | **0.854** |
| single+double+triple | 0.859 | 0.830 | **0.861** |
| AVERAGE | 0.832 | 0.742 | **0.834** |

Table S4. **Detailed information on the proteins listed in the dataset of Tables 1-3.** The protein fitness classification and the number of sites being mutated of each protein.

| protein fitness classification | dataset | Mutants (number of sites being mutated) |
| --- | --- | --- |
| catalysis | B3VI55_LIPST | 6541 (1) |
|  | MTH3_HAEAESTABILIZED | 1957 (1) |
|  | KKA2_KLEPN | 5278 (1) |
|  | MK01_HUMAN | 5463 (1) |
|  | AMIE_PSEAE | 6289(1) |
|  | RASH_HUMAN | 3040 (1) |
|  | UBC9_HUMAN | 2281 (1) |
|  | BG_STRSQ | 3000 (1) |
|  | TRPC_THEMA | 1520 (1) |
|  | TIM_SULSO | 1520 (1) |
|  | P84126_THETH | 1520 (1) |
|  | BLAT_ECOLX | 5468 (1) |
| protein stability | PTEN_HUMAN | 3014 (1) |
|  | TPMT_HUMAN | 2659 (1) |
| peptide binding | DLG4_RAT | 1578 (1) |
|  | YAP1_HUMAN | 363 (1) |
|  | WW | 629(1), 9713(2) |
| protein binding | F7YBW7 | 38 (1), 499 (2), 2798 (3), 5859 (4) |
|  | IF1_ECOLI | 1439(1) |
|  | SUMO1_HUMAN | 1329 (1) |
|  | RL40B_YEAST | 1176 (1) |
| DNA binding | FOSJUN | 1215(1), 107618(2) |
|  | GAL4_YEAST | 1193(1) |
| RNA binding | RRM | 1064(1), 36522(2) |
|  | TDP43 | 965(1), 52272(2) |
| Ig-G binding | GB1 | 1026(1), 516433(2) |
| fluorescence | GFP | 1084(1), 12777(2), 12336(3), 9387(4),  6825(5), 4298(6), 2526(7), 1364(8), 627(9),  299(10), 118(11), 43(12), 23(13), 5(14), 2(15) |

Table S5. **Ablation study results.** Ablation study was performed in the testing when we removed each of the three modules in the integrated model. The average spearman correlation of all datasets shows that model including all the three components are the most accurate, and all three parts contribute positively to the performance of the integrated model, with the global encoder contributing the most.

|  | without structure module | without global encoder | without local encoder | SESNet |
| --- | --- | --- | --- | --- |
| BLAT_ECOLX | 0.899 | 0.303 | 0.872 | **0.903** |
| P84126_THETH | 0.849 | 0.399 | 0.840 | **0.858** |
| RL40B_YEAST | 0.829 | 0.344 | 0.769 | **0.872** |
| TIM_SULSO | 0.785 | 0.228 | 0.765 | **0.790** |
| TRPC_THEMA | 0.769 | 0.436 | 0.780 | **0.783** |
| YAP1_HUMAN | 0.756 | 0.189 | 0.769 | **0.775** |
| DLG4_RAT | 0.722 | 0.157 | **0.782** | 0.748 |
| BG_STRSQ | 0.427 | 0.198 | 0.540 | **0.747** |
| IF1_ECOLI | 0.717 | 0.224 | 0.679 | **0.726** |
| PTEN_HUMAN | 0.668 | 0.318 | 0.637 | **0.715** |
| UBC9_HUMAN | 0.638 | 0.255 | 0.635 | **0.710** |
| RASH_HUMAN | 0.637 | 0.246 | 0.653 | **0.656** |
| AMIE_PSEAE | 0.629 | 0.203 | **0.640** | 0.632 |
| SUMO1_HUMAN | 0.592 | 0.294 | 0.591 | **0.609** |
| TPMT_HUMAN | 0.535 | 0.245 | 0.541 | **0.579** |
| GAL4_YEAST | 0.543 | 0.210 | **0.563** | 0.561 |
| MK01_HUMAN | 0.392 | 0.125 | 0.486 | **0.491** |
| KKA2_KLEPN | 0.435 | 0.120 | 0.446 | **0.446** |
| MTH3_HAEAESTABILIZED | 0.394 | 0.252 | 0.409 | **0.430** |
| B3VI55_LIPST | 0.390 | 0.193 | 0.386 | **0.412** |
| AVERAGE | 0.630 | 0.247 | 0.639 | **0.672** |

Table S6. **Ablation study of the pre-trained model** **tested on GFP datasets.** The spearman correlation was predicted by our models which is pre-trained on single-site and numerous double-sites variants generated by the unsupervised model ESM-IF1.

|  | without global encoder  and structure module | without local encoder  and structure module | SESNet |
| --- | --- | --- | --- |
| 2 sites | 0.458 | 0.389 | **0.463** |
| 3 sites | 0.453 | 0.447 | **0.538** |
| 4 sites | 0.381 | 0.499 | **0.608** |
| 5 sites | 0.295 | 0.537 | **0.657** |
| 6 sites | 0.217 | 0.555 | **0.683** |
| 7 sites | 0.163 | 0.566 | **0.698** |
| 8 sites | 0.130 | 0.570 | **0.704** |
| 9 sites | 0.113 | 0.573 | **0.707** |
| 10 sites | 0.104 | 0.574 | **0.708** |
| 11 sites | 0.100 | 0.574 | **0.709** |
| 12 sites | 0.099 | 0.575 | **0.709** |
| AVERAGE | 0.228 | 0.533 | **0.653** |

Table S7. Hyperparameter configurations for different dataset.

|  | single-site datasets | multiple-sites dataset |
| --- | --- | --- |
| Hidden-size | 256 | 512 |
| Mini-batch size | 32 | 32 |
| Learning rate | 5e-4 | 5e-5 |
| Warmup steps | 2000 | 2000 |
| Dropout | 0.2 | 0.2 |
| Max epochs | 1500 | 1500 |
| Patience for early stop | 150 | 150 |


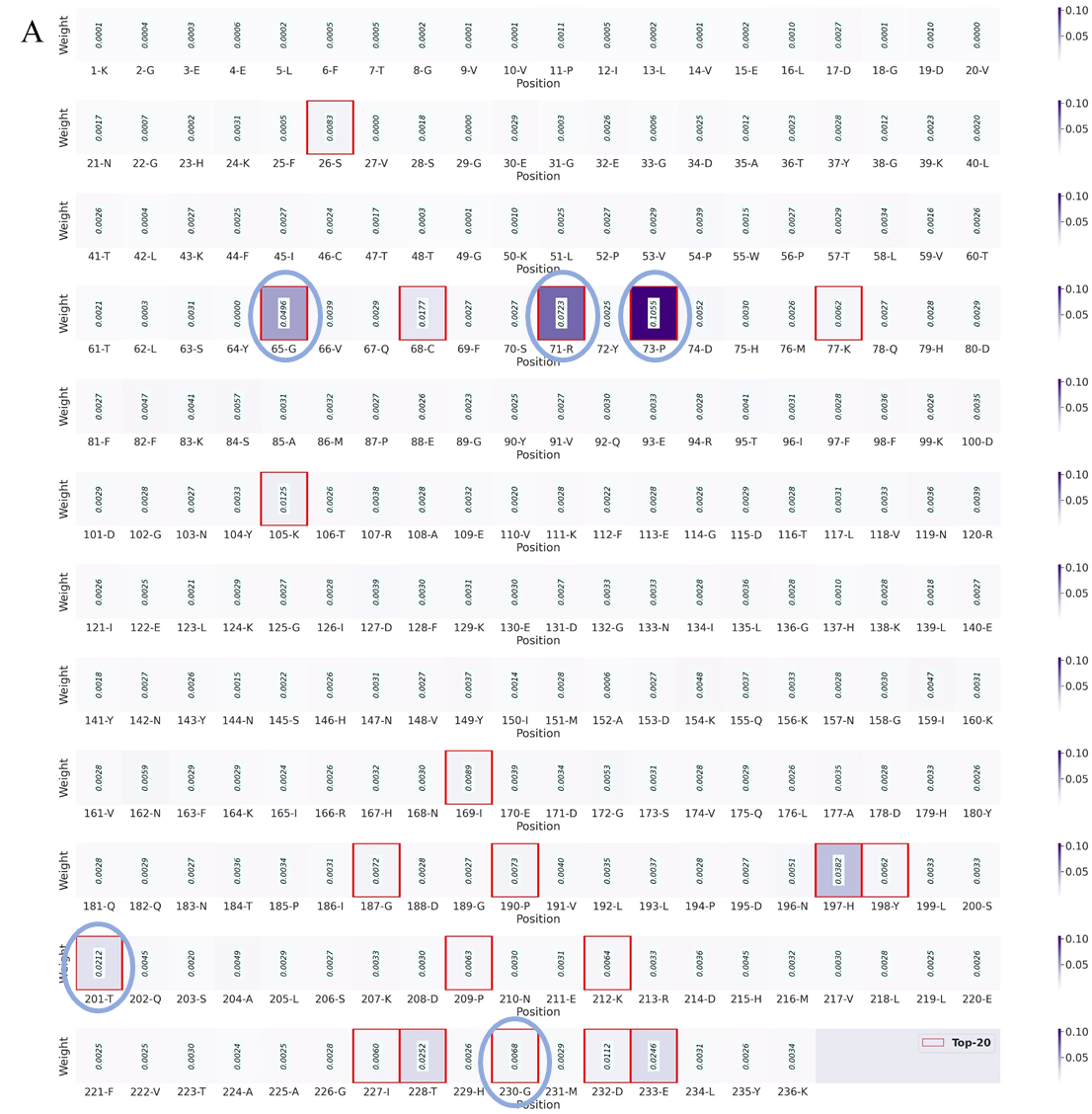


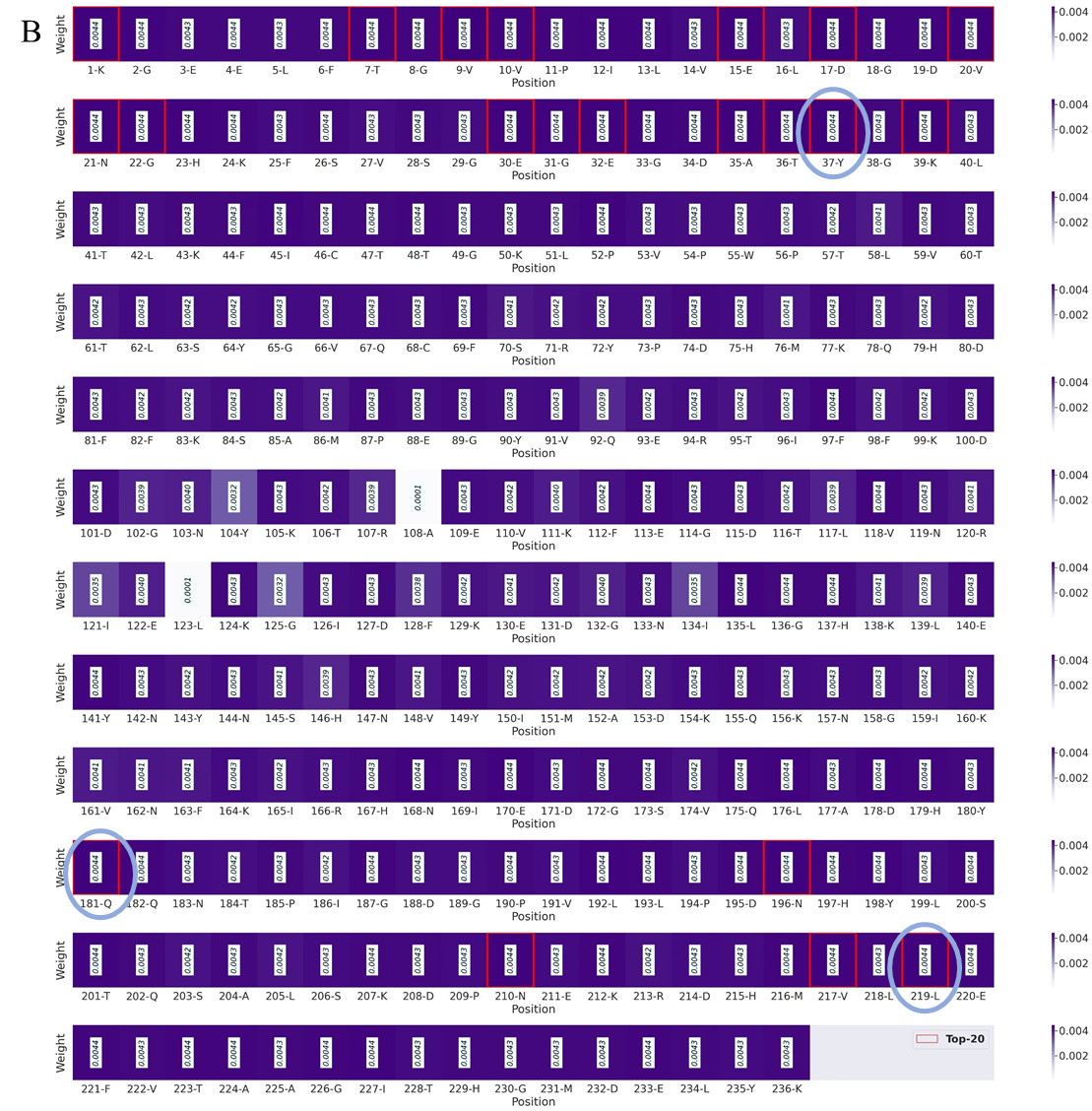


Figure S1. **Attention score of sites on the wildtype sequence.** Attention scores of sites generated by SESNet (A) and the model without the structure module (B) trained on the 1084 single-site mutants of the dataset of GFP. We picked up the top 20 attention-score AA sites predicted by SESNet with and without structure module, respectively. When the structural module is present, there are five sites (marked by the blue ellipse in the subgraph A) identified by our model accords with the key AA sites discovered by experiments (mentioned in main text). However, this number is reduced to three when we remove the structural module from the model (marked by the blue ellipse in the subgraph B).


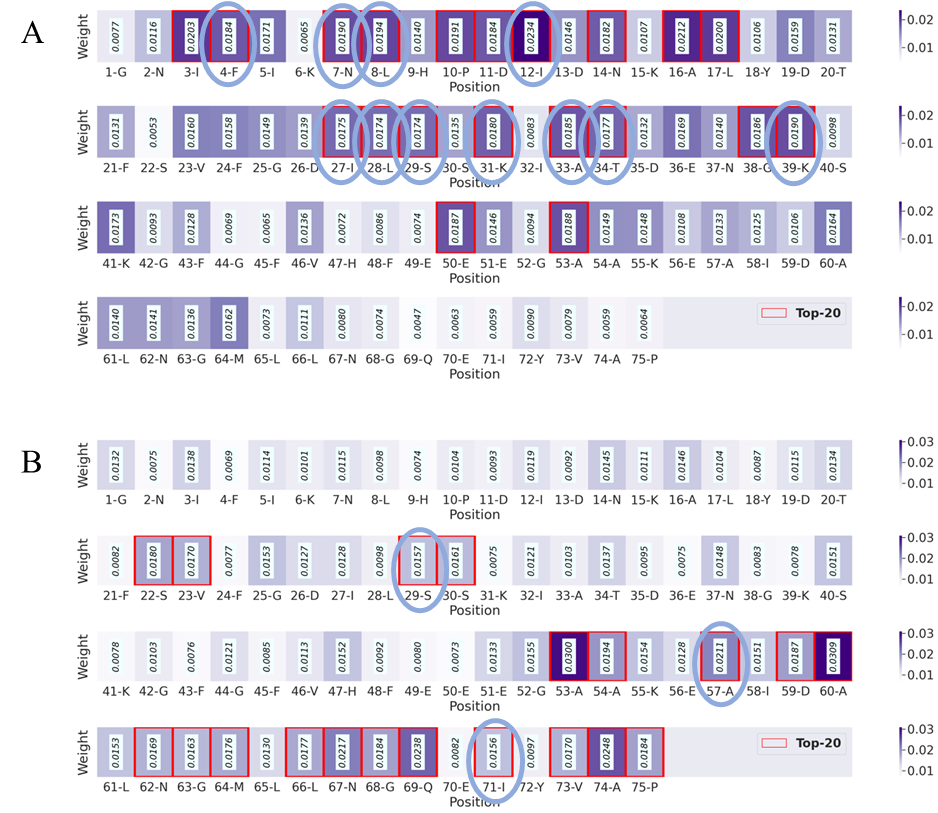


Figure S2. **Attention score of sites on the wildtype sequence.** Attention scores of sites generated by SESNet (A) and the model when removing the structure encoder (B) trained on the 1064 single-site mutants of the dataset of RRM. We picked up the top-20 attention-score AA sites predicted by SESNet model with and without the structure module, respectively. When the structural module is present, there are 11 sites (marked with blue ellipse in the subgraph A) predicted by the model accord with the key AA sites discovered by experiments (mentioned in main text). In contrast, when removing the structure module, 3 of the predicted top-20 AA sites accord with the experimentally discovered (see the subgraph B).


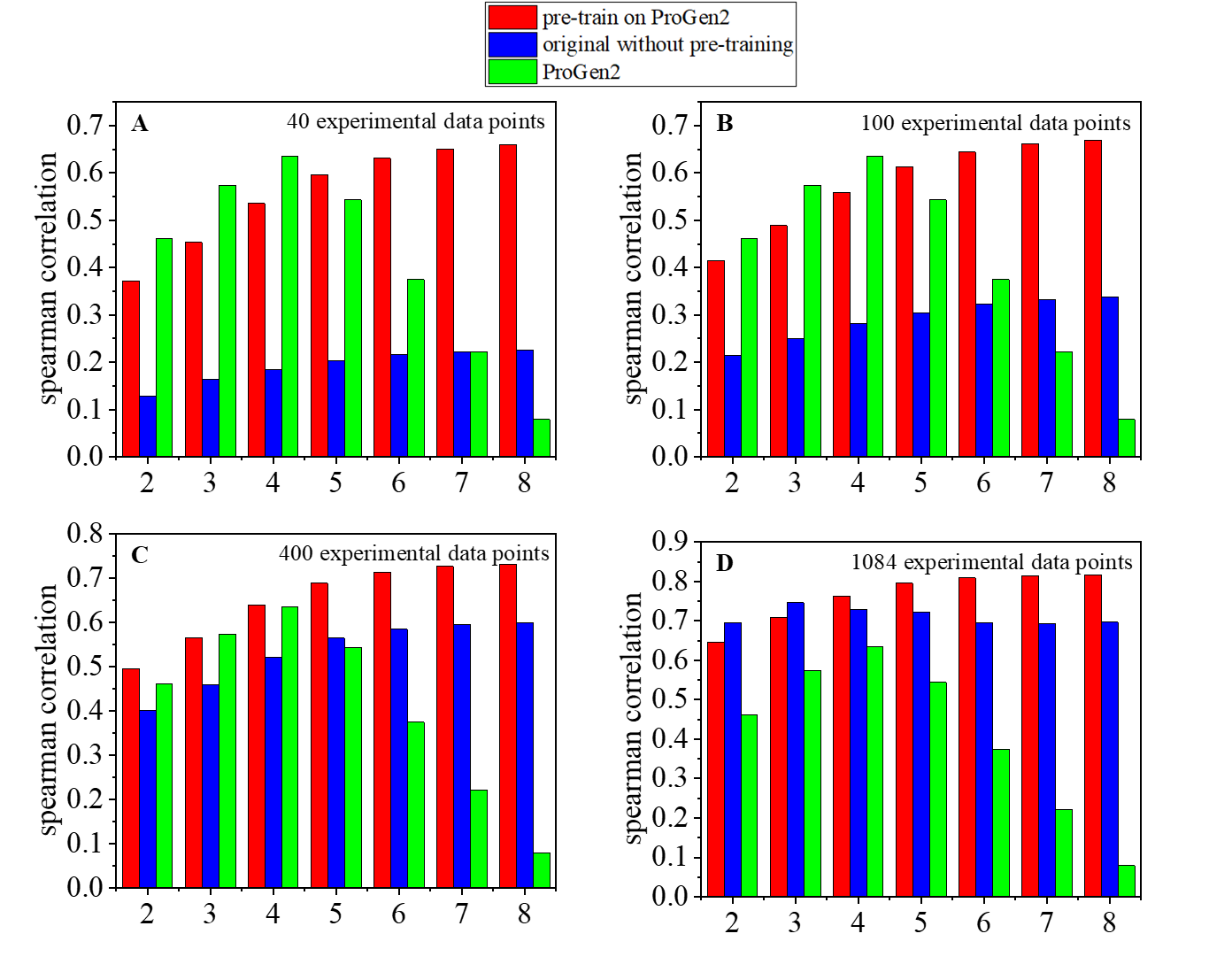


Figure S3**.** **Results of models pre-trained on the dataset generated by the unsupervised model** ProGen2 (ref), and then fine-tuned **on different number of experimental data points.** A-D: The spearman correlation of fitness prediction on multiple sites (2-8 sites) mutants by finetuning on 40, 100, 400, 1084 experimental single-site variants from dataset of GFP. Here, the red and blue bars represent the results of the model with and without pre-training, respectively. And the green bars correspond to the results of the unsupervised model ProGen2 as a control.


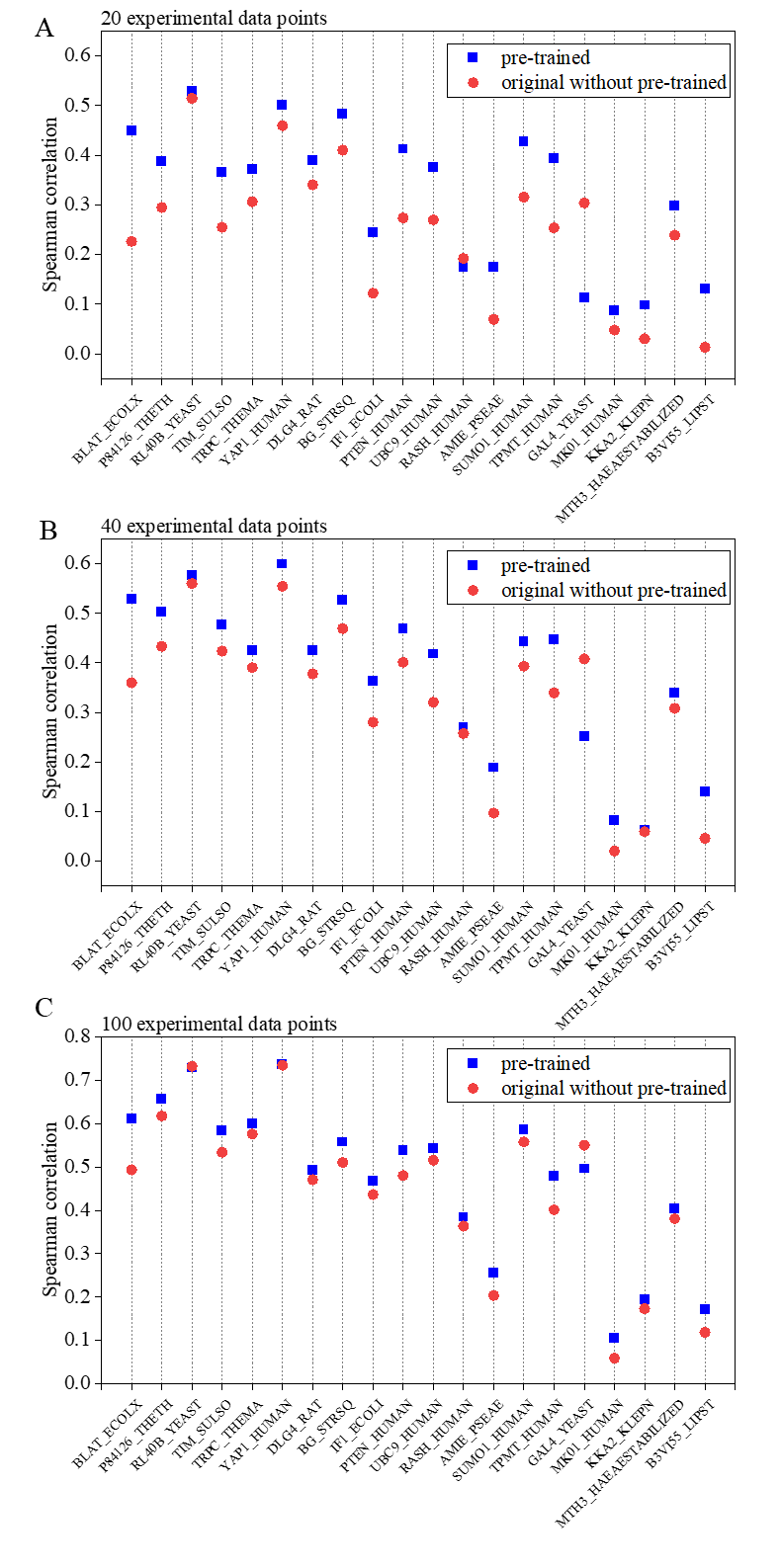


Figure S4**.** **Results of models trained on different number of single-site experimental variants.** A-C: The spearman correlation of fitness prediction on single-site mutants by finetuning on 20, 40, 100 single-site variants from different datasets. Where the blue and red symbols represent the results of the pre-trained model and the original model without pretraining, respectively.


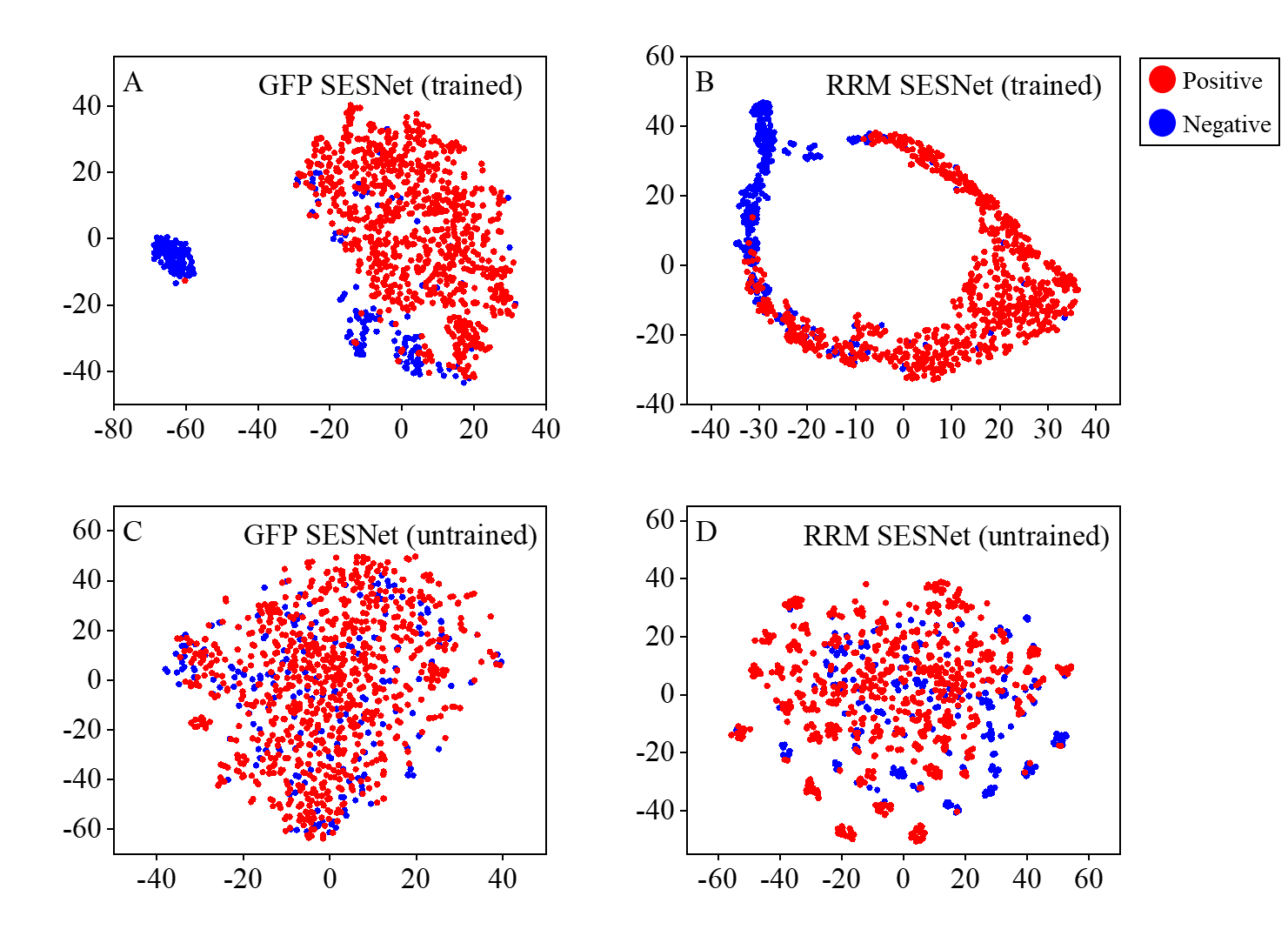


Figure S5**. Variant sequence representations of trained and untrained SESNet by the experimental data.** Each point represents a variant, where the positive and negative variants are colored as red and blue, respectively. The models were trained on single-site mutants from the dataset of GFP and RRM. Here a red point represents a mutant whose experimental fitness value is higher than that of the wild type, while the blue point gives mutant whose experimental fitness value is lower than the wild type. As can be seen in A and B, after training by part of the experimental data set, the positive and negative mutants can be separated into different spaces. In contrast, those representations from untrained model with random parameter initialization (C and D) do not reflect any clear separation between the positive and negative mutants as expected. This comparison shows that our model can learn to distinguish functional fitness of mutants into a latent representation space with supervised training.

Figure S6**. Representations of variants in different training ways.** A: The representations from the pre-trained models without fine-tuning by any experimental data. B: the representations from the pre-trained models, which is further finetuned on 40 single-site experimental mutants. C: the representations from the model directly trained on 40 single-site experimental mutants without pre-training.
